# Implementation and effectiveness of transgender stigma reduction interventions in sub-Saharan Africa: a scoping review

**DOI:** 10.29392/001c.72080

**Published:** 2023-03-24

**Authors:** Patience A. Muwanguzi, Paul K. Otiku, Racheal Nabunya, Blessings Gausi

**Affiliations:** 1 College of Health Sciences, School of Health Sciences, Makerere University, Uganda; 2 Department of Population Health, Azrieli Faculty of Medicine, Bar Ilan University, Israel; 3 School of Health Sciences, College of Health Sciences, Makerere University, Uganda; 4 School of Public Health and Family Medicine, Faculty of Health Sciences, University of Cape Town, South Africa

**Keywords:** Transgender, sub-Saharan Africa, stigma

## Abstract

**Background:**

The transgender or trans population is one of the most marginalized social groups globally, frequently experiencing ill-treatment and discrimination. This is disproportionately higher in sub-Saharan Africa where trans people experience stigma even in healthcare settings. There is limited evidence concerning the implementation and outcomes of interventions to mitigate this stigma. Therefore, this scoping review aimed to describe interventions and determine their effectiveness in reducing transgender stigma in sub-Saharan Africa.

**Methods:**

Searches (completed November 01, 2021, and re-run May 2022) were conducted in MEDLINE (via PubMed), Cochrane Library including the Cochrane Central Register of Controlled Trials, EBSCOhost, Cumulative Index to Nursing and Allied Health Literature (CINAHL), Embase, Web of science, clinicaltrials.gov, and online grey literature sources to identify publications that described interventions to reduce transgender stigma in sub-Saharan Africa.

**Results:**

From 877 literature search results, 23 full-text articles were assessed. Data were extracted from the four (4) eligible papers. Only one study explicitly mentioned transgender people. Second, while two studies incorporated conceptual frameworks, they did not show how the frameworks guided the study. The four studies implemented unique interventions at various socio-ecological levels to address individual and interpersonal and structural stigma. Each study utilized a different methodological approach, and the interventions were all evaluated qualitatively.

**Conclusions:**

There is a paucity of transgender stigma reduction interventions implemented in Sub-Saharan Africa with limited evidence of interventions delivered to mitigate stigma at interpersonal and structural levels. Future anti-transgender stigma research should consider reporting details about the core components and descriptions of the interventions. Additionally, the use of validated measures of stigma and the evaluation of interventions for implementation outcomes would be helpful.

The term ‘transgender’ describes persons who do not identify with the sex category assigned to them at birth, or whose gender identity is incongruent with gender norms.^1,2^ The transgender or trans population is one of the most marginalized social groups globally, frequently experiencing maltreatment and discrimination.^3^ This mistreatment and discrimination are termed *transphobia*, described as an irrational fear and hatred of transgender persons because they do not conform to societal gender norms.^4,5^ Herek defines gender minority stigma as “stigma directed at non-normative gender identities, experiences, and expressions as well as gender minority communities”.^6^ Stigma occurs when elements of labelling, stereotyping, separation, status loss, and discrimination occur together in a power situation that allows them.^7,8^

Anti-trans or transgender stigma runs the gamut from experienced or enacted stigma, to felt, perceived, or anticipated stigma, and to internalized or self-stigma.^6^ The complexity of stigma raises challenges around measurement including the level, severity, and frequency.^9^ Transgender stigma displays similar intricacy by working through multi-faceted risk factors to influence numerous health outcomes.^10,11^ Several health researchers have used the socioecological framework to elucidate that stigma operates at multiple levels to impact health.^12–14^ Trans women particularly face “systemic oppression” or “state-sanctioned stigma” codified in laws or rooted in societies’ normative beliefs in addition to interpersonal and individual forms of stigma.^15–17^ Stigma in low-resource settings tends to be a greater obstacle to accessing services than in other contexts.^18^ Healthcare-related stigma goes beyond the avoidance of healthcare services, but the added discomfort and stress may negatively affect health.^19,20^ For instance, structural, interpersonal, and individual forms of stigma have been linked to adverse health outcomes including depressive symptoms, anxiety, suicidality, substance abuse, condomless sex and HIV among transgender people.^21–23^ The gender minority stress theory suggests that added stressors related to the stigma attached to one’s incongruous gender identity/expression adversely affect health and accounts for health differences between transgender and cisgender individuals.^24^ The core of transgender individuals’ views on healthcare challenges is gender insensitivity, displays of discomfort, denied services, suboptimal or forced care, and verbal abuse.^25^

The challenges faced by African transgender populations are exacerbated by the criminalization of lesbian, gay, bisexual, and transgender (LGBT) communities in most African countries.^26–30^ In South Africa, transgender people experienced stigma while accessing reproductive health care,^3^ while other transfeminine and gender diverse women faced stigma and discrimination due to their gender identity and gender expression.^31^ The participants reported encounters with hostile health services, and that all unrelated health problems were turned into problems concerning gender or sexual identity, causing discomfort for the patient.^3^ In Uganda, transgender women reported different forms of stigma including beatings by the police and by sex work clients, abuse and discrimination received in the workplace or from family members, and the lack of tailored health services.^16^ A study in Kenya, Malawi, and South Africa found that nearly half of the men who have sex with men (MSM) and transgender women (TGW) who participated (45.3%) reported at least one healthcare-related stigma experience. The most frequently reported healthcare-related stigma experience was feeling afraid to seek healthcare services (36.3%).^19^ In Uganda trans-women sex workers reported internalized stigma as one of the key barriers to the access and utilization of HIV/STI prevention and care services.^32^ In Rwanda, a study among MSM and TGW reported that anticipated, perceived, and enacted stigmas were highly prevalent, and significantly higher among trans-women (*p* < 0.001), and demonstrated a high burden of depressive symptoms and depression among MSM/TGW in Kigali.^33^

Stigma reduction or alleviation interventions have been developed to improve coping at the individual level, reduce the enactment of stigma at the interpersonal level, and change the norms, policies, and systems that propagate stigma at the structural level.^12^ The availability of data on health-related stigma and discrimination is critical for improving interventions and programs to address them, yet such routine data are often lacking.^13^ There is limited literature regarding transgender stigma in sub-Saharan Africa and even less evidence concerning the implementation and outcomes of interventions to mitigate this stigma. Therefore, the objective of this scoping review was to assess the extent of the literature on the implementation and effectiveness of transgender stigma reduction interventions in sub-Saharan Africa.

## METHODS

The scoping review methodology was selected since the aim of this study was to map existing evidence, and identify gaps and the main sources and types of evidence available.^34^ Additionally, we chose the scoping review methodology given that the topic has not been fully explored. This review provides synthesized evidence that health policymakers and programs in similar settings may be use to design evidence-based stigma reduction interventions to improve healthcare access for transgender people and other sexual and gender minority populations.

The review was guided by the JBI methodology for scoping reviews^35^ and the framework initially described by Arksey and O’Malley^34^ and modified by Levac et al.^36^

### STEP 1: IDENTIFYING THE RESEARCH QUESTION

The research question that guided this scoping review was “*What is the implementation and effectiveness of transgender stigma reduction interventions in sub-Saharan Africa?*

Specifically, the review seeks to answer the following questions:

1. Who is the target population for transgender stigma reduction interventions?
2. What type of interventions are being implemented to reduce transgender stigma?
3. What are the outcomes of the interventions aimed at reducing transgender stigma?
4. What is the effectiveness of stigma reduction interventions in reducing or alleviating transgender stigma?

### STEP 2: IDENTIFYING RELEVANT STUDIES

The search strategy aimed to locate both published and unpublished studies. Before commencing the search for relevant studies, we sought advice from one of the medical librarians in the college of Health Sciences at Makerere University.

An initial limited search of MEDLINE was undertaken to identify articles on the topic. The text words contained in the titles and abstracts of relevant articles, and the index terms used to describe the articles were used to develop a full search strategy. The search strategy was applied to different databases by the Peer Review of Electronic Search Strategies guidelines.^37^ We conducted the pilot exercise by running searches on the primary database (MEDLINE). Search terms and free-text words were combined using the Boolean operators ‘AND’ and ‘OR’. Search terms also included other controlled descriptors such as Medical Subject Headings and their synonyms. We modified the search strategy and research question after greater knowledge of the literature was acquired. The search strategy was adjusted to cater to differences in functionality of other databases. (Online Supplementary Document, Table S1)

A broad literature search was carried out on the following electronic databases: MEDLINE (via PubMed), Cochrane Library including the Cochrane Central Register of Controlled Trials, EBSCOhost, Cumulative Index to Nursing and Allied Health Literature (CINAHL), Embase, Web of science, clinicaltrials.gov, and the International Clinical Trials Registry Platform. Additionally, we searched the internet for articles that could be accessed through networks and organizations, including articles known to the authors. We identified additional literature by hand searching related journals and reference lists of relevant articles. Relevant grey literature was sourced from Mednar for potentially eligible articles and conference proceedings, white papers, and theses. We employed no language restrictions during the search and screened any article published from database inception to the index search date (01 November 2021). We re-run the search in May 2022.

After obtaining full texts, potentially eligible articles were screened at two levels: title and abstract screening and full-text screening each time, selecting eligible studies based on pre-determined inclusion/exclusion criteria. Two reviewers independently screened the titles and abstracts of all retrieved records from the search output (PAM, RN). Articles meeting the inclusion criteria were further subjected to a full-text assessment for eligibility. Disagreements between the reviewers on the articles to include were solved through consensus.

### STEP 3: STUDY SELECTION

We applied the PCC (Population, Concept, and Contexts) framework to define the inclusion criteria as proposed by Peters et al.^38^

#### Population.

Populations eligible for this scoping review included all categories of individuals involved in providing or receiving transgender healthcare, including transmen, transwomen, gender-diverse persons, gender non-conforming persons, staff at community-based organizations, staff at drop-in centres, health workers, peer educators, peer counsellors, partners of transgender people, among others.

#### Concepts.

The concepts related to the stigma reduction intervention were assessed following the template for intervention description and replication (TIDier) guidelines.^39^ These included the type of intervention, mode of delivery such as face-to-face, setting of intervention, implementers of the intervention, timing or duration or dose, materials or information given to participants, procedures, activities and/or processes of the intervention including enabling and support activities, tailoring of the intervention, modifications during the study, if intervention fidelity or adherence were assessed, and how the intervention outcomes were determined.

#### Context.

We limited the literature search to studies conducted in Africa. Therefore, eligible studies fulfilled the following criteria:

1. Reported stigma reduction among transgender persons, including studies on lesbian gay bisexual, and transgender (LGBT) populations or key populations that specifically mention transgender persons among those populations. Studies that examined stigma using samples of trans and cisgender (i.e., non-transgender) participants needed to present disaggregated data on the trans-sub-sample to be included.^40^
2. Reported stigma reduction intervention(s)
3. Reported quantifiable outcome(s) of stigma reduction intervention(s)
4. Were conducted in Africa

This scoping review considered both experimental and quasi-experimental study designs including randomized controlled trials, non-randomized controlled trials, hybrid trials, before and after studies, and interrupted time-series studies. In addition, analytical observational studies including prospective and retrospective cohort studies, case-control studies, and analytical cross-sectional studies, with or without comparator groups were considered for inclusion. This review also considered descriptive observational study designs including case series, individual case reports, and descriptive cross-sectional studies for inclusion.

Qualitative studies that focused on qualitative data including, but not limited to, designs such as phenomenology, grounded theory, ethnography, qualitative description, action research, and feminist research were also included. In addition, systematic reviews that met the inclusion criteria were considered, depending on the research question. We excluded studies with intervention or goals that were related to stigma reduction in general without a focus on transgender people.

Text and opinion papers were also considered for inclusion in this scoping review.

### STEP 4: CHARTING THE EVIDENCE

Following the search, all identified citations were collated and uploaded into EndNote version X9/2018 (Clarivate Analytics, PA, USA), and duplicates were removed. For each eligible study under consideration, one of three authors (PAM, RN, VKM) independently abstracted and recorded the data from eligible articles using a data abstraction tool containing study and intervention characteristics onto a shared spreadsheet. The abstraction tool included five domains: (1) study details (2) design and methods (3) type of stigma targeted (4) Intervention and outcomes and (5) key findings. The five domains are presented in table 1 adapted from Kemp et al.^41^ The full text of selected citations was assessed in detail against the inclusion criteria by two independent reviewers. Reasons for exclusion of sources of evidence at the full-text level that did not meet the inclusion criteria were recorded and are reported in the scoping review.

Two additional reviewers double-checked the entered data independently for completeness and verified the accuracy of the analysis. Disagreements that arose between the reviewers at each stage of the selection process were resolved through discussion, or with an additional reviewer as needed. Two other review team members checked the data for accuracy and fidelity to the study protocol. For validity, an independent medical librarian re-ran the search strategy on the different databases and followed the same process to confirm the findings.

### STEP 5: SYNTHESIZING AND REPORTING THE EVIDENCE

The reporting of the findings of this review follows the Preferred Reporting Items for Systematic reviews and Meta-Analyses extension for Scoping Reviews guidelines.^43^ (Online Supplementary Document, Table S2)

Data were extracted from papers included in the scoping review by two or more independent reviewers using a data extraction tool developed by the reviewers. The data extracted included specific details about the participants, concept, context, study methods, and key findings relevant to the review question/s. A pre-tested extraction form in MS Excel was used by the reviewers. Any disagreements that arose between the reviewers were resolved through discussion. Two authors of papers were contacted to request additional data.

#### Analysis.

For qualitative studies, the data were recorded and summarized narratively. Key themes or a summary of relevant results were extracted. Any contextual factors identified to affect stigma reduction were also extracted. Quantitative evidence was aggregated and summarized using appropriate statistics and methods. A narrative synthesis of key results was conducted for each stigma reduction intervention and outcome. No meta-analysis of critical appraisal was performed.

### STEP 6: CONSULTATION WITH STAKEHOLDERS

We engaged key stakeholders from transgender-led community-based organizations in sub-Saharan Africa, health care workers at trans-friendly health facilities, policymakers, psychologists, and experts in HIV programming among others to obtain relevant grey literature that may not have been captured by the literature search. Some of these stakeholders also provided support with the identification of critical concepts and in the contextual interpretation of the review findings. The stakeholders will receive a copy of the review findings to read through and assess whether the review has captured the critical concepts and interpreted them correctly in the context of transgender stigma. They will then provide their comments and suggestion directly in the review document.

## RESULTS

### SCREENING RESULTS

**Figure 1.**
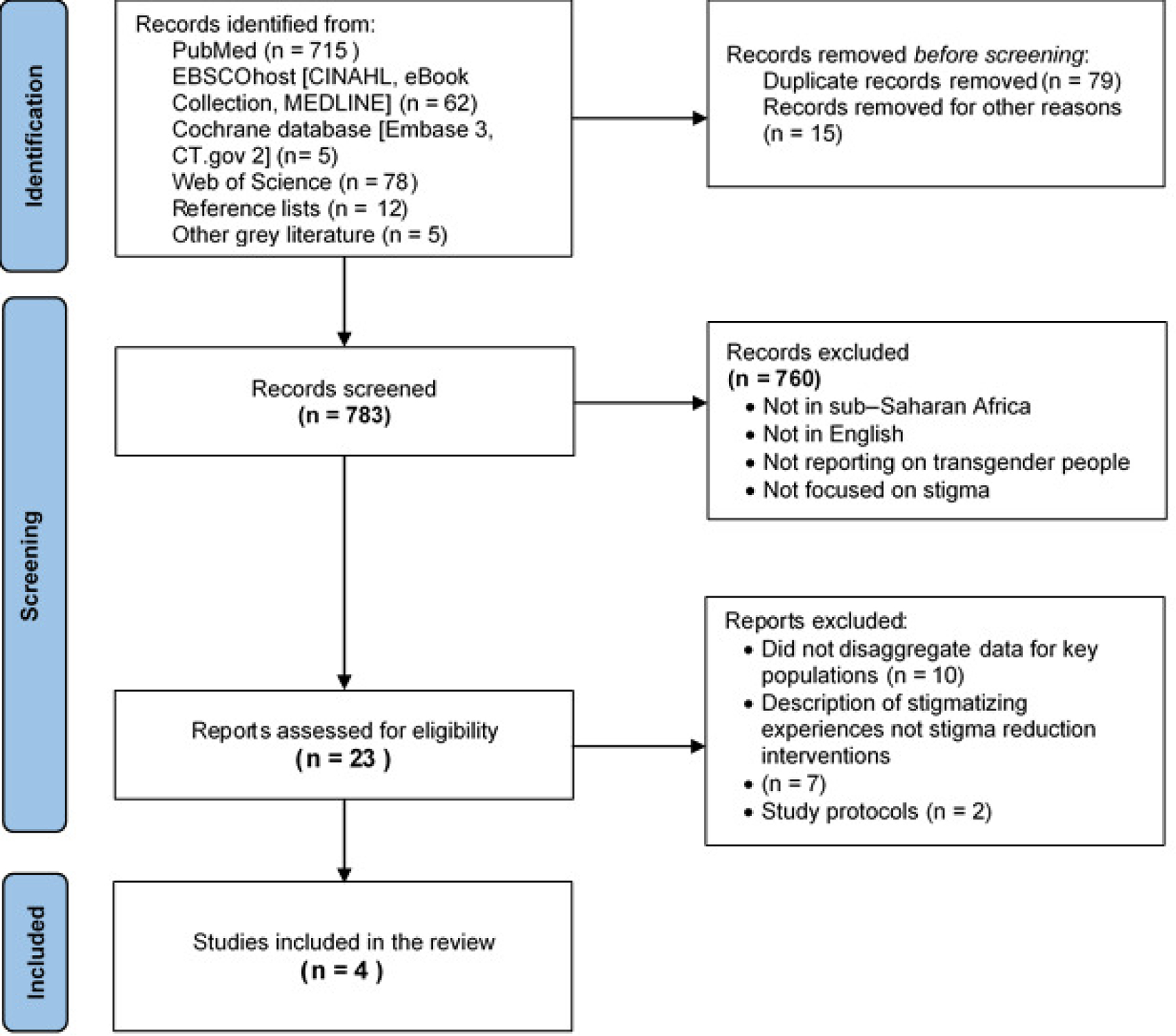
PRISMA flow chart of search

We screened 877 studies and assessed 23 full-text articles for eligibility. A total of 4 studies met all the inclusion criteria^17,44–46^ (Figure 1) and they implemented a range of stigma reduction interventions. Several studies were excluded from the review because while they conducted stigma reduction interventions among key populations, they did not disaggregate the data for transgender people.

### CHARACTERISTICS OF INCLUDED STUDIES

All the included studies were published between 2019 and 2022, resulting in a total sample size of 2526 participants from primary studies. They were conducted in countries in Sub-Saharan Africa, with one multi-study that conducted interventions in two other Caribbean countries.^17^ Regarding the study type, one was qualitative,^44^ one was a pre-and post-test intervention cross-sectional survey,^45^ and one was a qualitatively driven triangulated longitudinal mixed-methods design.^17^ Of the studies included, only one utilized a conceptual framework: community-based research (CBR).^44^ The study participants included community stakeholders,^44^ clinical and non-clinical staff^45^ and organizations for advocacy of LGBTQI and other key stakeholders.^17^ One study explicitly mentioned transgender people among the study participants,^44^ while another involved SGM peer educators.^46^ Another study did not report demographic information about study participants for their security, therefore the involvement of transgender people is not clear.^17^ Only one study utilized a globally validated tool measuring health worker stigma and discrimination.^45^ Table 2 illustrates the characteristics of the included studies.

### THE GEOGRAPHICAL LOCATION OF INCLUDED STUDIES

**Figure 2.**
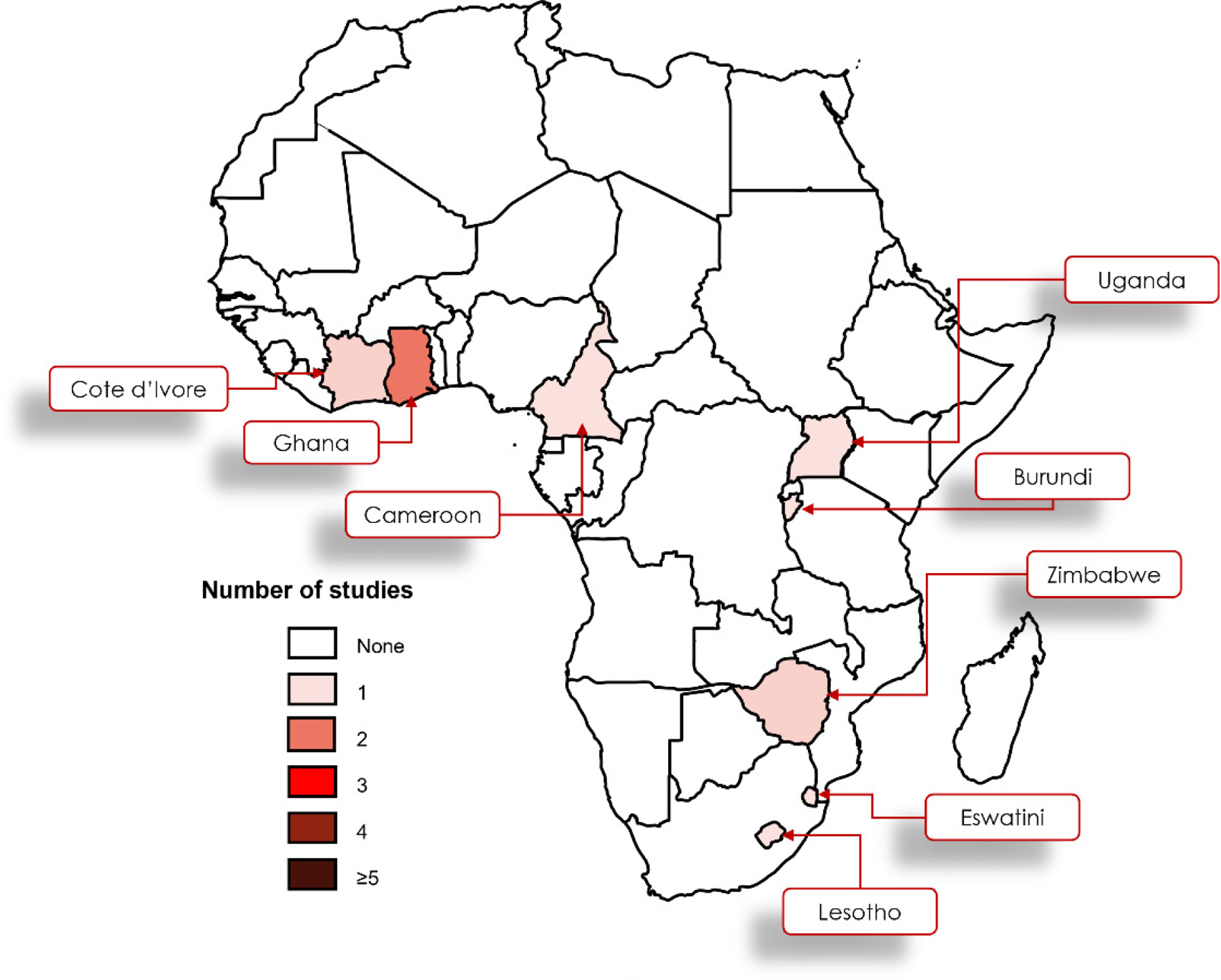
Frequency distribution of included studies

The studies were conducted in Swaziland (Eswatini) and Lesotho,^44^ Uganda,^46^ Ghana,^45^ and Ghana, Burundi, Cameroon, Côte d’Ivoire, and Zimbabwe^17^ (Figure 2).

### INTERVENTION LEVEL DESCRIPTIVE CHARACTERISTICS

The intervention in Lesotho and Eswatini (Swaziland) entailed a participatory theatre intervention (PTI) that involved two components and targeted interpersonal stigma from different community stakeholders.^44^ The Ghana “total facility” intervention was a two-day participatory stigma-reduction training for all staff levels with staff and clients who were trained as stigma-reduction facilitators. The intervention targeted interpersonal stigma and focused on observed and perceived stigma among clinical and non-clinical staff.^45^ The MARPI model training in Uganda was conducted by SGM peer educators for research staff for a clinical trial, about inclusive service delivery.^46^ Finally, advocacy and other community tactics (ACT) intervention targeted structural stigma in five African countries and self-stigma in Ghana, through partnerships with stakeholders for advocacy.^17^ Table 3 illustrates the intervention level characteristics of the included studies.

### IMPLEMENTATION AND EFFECTIVENESS OUTCOMES AND FINDINGS

The participatory theatre intervention led to an improvement in attitudes and an understanding of the perceptions of the negative impacts of LGBT stigma. The key recommendations included expanding the interventions to targeted groups and rural communities.^44^ The total facility intervention reported a reduction in associated stigmatizing behaviour such as double gloving and fear of acquisition of HIV from clients. They recommended the utilization of implementation science approaches to improve effectiveness and sustainability.^45^ The MARPI-led intervention in Uganda resulted in SGM community partner’s anecdotal reports of a welcoming and inclusive environment. Following the training for the research team, the clinical trial was completed with 90% retention rates for MSM and 81% retention for transgender women at 12 months. They suggest expanding this to rural facilities using online and no-cost web-based resources.^46^ Finally, the advocacy and other community tactics multi-country intervention reported outcomes related to increased commitments to equality in access to HIV care and enhancement in an advocacy capacity. They recommend combining interventions targeting stigma at different levels for instance structural stigma and self-stigma for the LGBT persons.^17^ There were also undesirable outcomes among people who participated in LGBT advocacy including loss of income and stigma.^17^

## DISCUSSION

Mapping the evidence on evidence-based interventions for transgender stigma mitigation in sub-Saharan Africa is critical to inform the development of implementation strategies to deliver these interventions to the people who need them, to target different levels of stigma.

We reviewed the literature on the implementation and effectiveness of transgender stigma reduction interventions in sub-Saharan Africa. Twenty-three (23) full-text articles were eligible for analysis, with ten of these evaluating stigma reduction interventions among LGBT or key populations without disaggregating the data for transgender populations and were therefore not included in the final assessment. Various interventions were identified at various socio-ecological levels to address individual stigma and interpersonal and structural stigma. Each study utilized a different methodological approach, and the interventions were all evaluated qualitatively. None of the included studies targeted stigma among transgender people.

A detailed assessment of these studies suggested three key gaps in the literature. First, only one study in the sample explicitly mentioned transgender people. Second, while two studies incorporated conceptual frameworks, they did not show how the frameworks guided the study. Proctor and colleagues,^49^ proposed outcomes for assessing implementation research; acceptability, feasibility, adoption, appropriateness, cost, fidelity, penetration, or sustainability. All the studies did not assess these implementation outcomes. Third, while the studies described the interventions, only Nyblade and colleagues in Ghana^45^ provided sufficient details necessary for the potential replication and adoption of those interventions in other contexts. These details were however presented in other documents which are freely accessible online.

This scoping review identified 3 articles that were eligible for data extraction, published between 2019–2022, despite no time restrictions placed during the literature search phase. Therefore, this is a phenomenon that is just now coming into the limelight. Our findings demonstrate a gap in the literature on transgender stigma reduction interventions in SSA, which highlights the need for further implementation of science research to design or adapt evidence-based interventions. Two of the studies collected data over time, while another was a cross-sectional study. The studies however recognized the limitation of the short time frame of the evaluations which did not allow for the assessment of longer-term intervention effects.^44,45^ This raises the challenge of sustainability, considering that of the EBIs that are implemented, many are not continued after a certain amount of time.^50^ Sustainability is defined as “the extent to which an evidence-based intervention can deliver its intended benefits over an extended period after external support… is terminated”^51^ (p. 26).

The interventions in the three studies targeted stigma at different levels including structural stigma,^17^ interpersonal stigma by training health workers^45^ and participatory theatre intervention with key stakeholders like nursing students, the police and community leaders among others.^44^ Several studies recommend targeting a range of actors and socio-ecological levels for mitigation interventions among transgender people.^12,52^ Therefore, future stigma reduction interventions in sub-Saharan Africa may consider multi-level strategies.

The main recommendations from the studies included designing interventions that are coupled with activities to address other forms of stigma.^17^ Another study recommended that stigma-reduction interventions should be accompanied by rigorous implementation science to ensure ongoing learning and adaptation to maximize effectiveness and long-term impact.^45^ Finally, expanding interventions to targeted groups such as LGBT for interventions among other stakeholders, integrating interventions into existing community events and consideration of mechanisms to address policy- and community-level changes (structural) in stigma to foster lasting attitudinal changes.^44^

### STRENGTHS AND LIMITATIONS

This study reaffirmed the utility of scoping reviews in highlighting the evidence gaps in each field. The scoping review revealed the paucity of literature on the implementation and effectiveness of transgender stigma reduction interventions in sub-Saharan Africa. This provides an opportunity for researchers to conduct empirical research and design more evidence-based interventions to mitigate transgender stigma in SSA, but also to develop implementation strategies for the delivery of the identified EBIs. We employed a systematic approach to the literature search, utilizing the PCC framework, however, there is a possibility that some articles may have been missed.

## CONCLUSIONS

The study highlighted the paucity of transgender stigma reduction interventions implemented in Sub-Saharan Africa with limited evidence of interventions delivered to mitigate stigma at interpersonal and structural levels. Considering the limited evidence of interventions and ongoing research on this topic, we recommend prioritization of primary research and interventions targeting transgender populations in Africa. In addition, future anti-transgender stigma research should report details about the core components and descriptions of the interventions. Furthermore, we recommend the use of validated measures of stigma, as well as the evaluation of interventions for implementation outcomes.

## Supplementary Material

Supplementary Table1 1

Supplementary Table 2

## Figures and Tables

**Table 1.** Abstraction tool of study and intervention characteristics

Domains	Constructs	Description	Reference
**Study details**	Year of Publication	-	-
	Country/setting	Should be in sub-Saharan Africa	-
	Study objective	-	-
**Design and Methods**	Study design	Quantitative, Qualitative or Mixed methods	-
	Study type	randomised controlled trials, pragmatic trials, non-randomised controlled trials, and observational studies, with or without controls, hybrid trials	-
	Study population	Community, transgender person, provider and/ or policy makers	-
	Hypothesis stated	-	-
	Conceptual model or theory used	Whether the intervention was motivated by a conceptual model, or the study is guided by a theory	-
	Validated measure(s)	Whether measures used were validated	-
	Sample size	-	-
	Sample characteristics	-	-
	Follow-up period	-	-
**Type of stigma**	Level of stigma targeted	Individual, interpersonal, and/ or structural	White Hughto et al.^12^
	Type of stigma targeted	Enacted, internalized, and/or anticipated	Earnshaw et al.^42^
	Measurement	How were stigma and stigma reduction measured?	-
**Interventions and outcomes**	Intervention name	The official name of the intervention has been stated	Hoffmann et al. (TIDieR checklist)^39^
	Intervention background	Rationale, theory, or goal of the elements essential to the Intervention	
	Intervention description	Mode of delivery, location/setting of intervention, Implementers of intervention, timing or duration or dose, materials or information is given to participants, procedures, activities and/or processes of the intervention including enabling and supporting activities, tailoring of intervention vs. homegrown, modifications during the study, if intervention fidelity or adherence were assessed	
	Level of stigma reduction intervention	Individual, interpersonal, and/ or structural	White Hughto et al.^12^
	Outcomes reported	-Implementation Outcomes-Acceptability, adoption, appropriateness, feasibility, fidelity, cost, penetration, and/or sustainability - effectiveness and outcomes related to stigma, or transgender health	Proctor et al.
**Key findings**	Conclusions and recommendations	-	-

**Table 2.** Summary characteristics of included studies

First author	Country	Study aim	Study design/type	Hypothesis tested	Study population	Sample size (n)	Validated stigma measure	Conceptual model used
Logie et al., 2019	Swaziland Lesotho	To understand the potential of participatory theatre as a mechanism to change stigmatizing attitudes towards LGBT people in Swaziland and Lesotho.	Qualitative	-	Community stakeholders-nursing students, nurses, educators, community leaders, police, and other community members	106 Transgender persons n=4	-	Community-based research (CBR)
Nyblade et al., 2020	Ghana	To evaluate the impact of a “total facility” stigma-reduction intervention on the drivers and manifestations of stigma and discrimination among health facility staff in Ghana.	Pre-and post-intervention cross-sectional survey	-	Clinical and non-clinical staff	2308 (1154 in each of the pre- and post-intervention periods)	Globally validated tool for health worker stigma and discrimination^47^	-
Miller et al., 2021	Burundi, Cameroon, Côte d’Ivoire, Ghana, Zimbabwe	To document the intended and unintended outcomes of an advocacy initiative designed to contribute to dismantling structural barriers to HIV care for gay and bisexual men and transgender women in select countries in Africa.	Qualitatively driven triangulated longitudinal mixed-method design	-	Lead collaborating partners, medical community, Government officials, Media, LGBTQI organizations, LGBTQI constituents, Other civil society organizations,	112	-	-
Keuroghlian et al., 2021	Uganda	To synthesize facilitators of and barriers to SGM health training efforts for healthcare workers in Uganda, to inform priorities, strategies, and next steps to advance culturally responsive HIV-related care for SGM communities.	Commentary	-	Clinical trial Research staff	-	-	-

**Table 3.** Intervention level descriptive statistics

First author	Intervention name	Intervention description	Level of stigma	Type of stigma	Key findings/Themes	Recommendations
Logie et al., 2019	PTI: Participatory Theatre Intervention	The PTI involved two components. Community animators from the theatre groups enacted the skits. The animators performed each play once to illustrate the situation and a particular experience of stigma. This initial enactment resulted in a crisis with no solution offered. Each play was performed a second time, and one of the co-facilitators stopped the play at a key point where there was a challenge and invited one or more of the intervention participants to portray a more positive and supportive solution. The participants, then acted out a possible solution.	Interpersonal	-	**1.** Understanding Perceptions of Negative Impacts of LGBT Stigma. **2.** Change in Attitude or Perspective Through Self-Reflection **3.** Change in Attitude or Perspective Through Learning **4.** Participatory Theatre Format as Supporting Change **5.** Ambivalence in Changing Attitude or Perspective	**1.** Expand interventions to targeted groups **2.** Integrate interventions into existing community events **3.** Focus interventions on rural communities **4.** consider mechanisms to address policy- and community-level changes in stigma to foster lasting attitudinal changes **5.** Social-ecological approaches to PTI to help identify barriers and facilitators to attitudinal change.
Nyblade et al., 2020	Ghana “total facility” intervention	Two-day participatory stigma-reduction training for all staff levels with delivery by staff and clients from the facilities who were trained as stigma-reduction facilitators. The training includes 14 core activities.^48^	Interpersonal	Observed and perceived	**1.** Fear of acquiring HIV while providing care for clients living with HIV improved significantly in the intervention versus comparison facilities, as did associated stigmatizing avoidance behaviors such as double gloving. **2.** No statistically significant Difference-in-differences between the intervention and control facilities on the composite stigmatizing attitudes variable.	Health facility stigma-reduction interventions should be accompanied by rigorous implementation science to ensure ongoing learning and adaptation to maximize effectiveness and long-term impact.
Miller et al., 2021	ACT: Advocacy and Other Community Tactics	**1.** Awareness building employing instructional videos, written educational materials, conferences, and other communication platforms **2.** Community mobilization **3.** Documentation involving systematic collection and reporting of data on violations **4.** Policy analysis and engagement **5.** Self-stigma reduction using small-group workshops and discussion forum **6.** Sensitization using small-group workshops and other training formats.	Structural	Ghana self-stigma	**1.** Increased commitments to Equality in Access to HIV Care **2.** improvements in access to HIV care through the creation of new resources or easing of access to existing resources **3.** enhancement in the advocacy capacity **4.** increases in the coverage and framing of issues pertinent to access to health care/human rights **5.** informal changes to exclusionary practices **6.** Formal Policy changes	**1.** Synergistic effects of structural inventions when combined should be further explored **2.** Structural interventions may benefit from being coupled with activities to address self-stigma and self-care
Keuroghlian et al., 2021	MARPI: (Most At Risk Populations Initiative) training model	Provider training in cultural responsiveness and involvement of SGM peer educators **1.** Equipping HIV and sexual healthcare staff with the knowledge, skills and empathy needed to serve SGM people **2.** Increasing understanding of societal stigma’s relationship to health disparities **3.** Cultivating staff’s insight into their own personal level of comfort and confidence serving SGM people **4.** Fostering vigilance to keep personal attitudes towards SGM people separate from professional behaviour **5.** Applying key concepts and terminology for sensitive and effective communication **6.** Promoting warmth and sincerity in service delivery **7.** Raising awareness of verbal and nonverbal communication **8.** Not trying to change people’s SGM identities and prioritizing flexibility, choice, and autonomy in care.	Interpersonal	-	**1.** Anecdotally, SGM community partners have reported that the research clinic is inclusive and welcoming. **2.** The clinical trial was completed with 90% retention rates for MSM and 81% retention for transgender women at 12 months.	**1.** Online live trainings and technical assistance programmes and no-cost web-based educational resources may facilitate the scale-up of tailored HIV and sexual healthcare for SGM communities in rural areas. **2.** Establish partnerships with local SGM communities
