# Supplementary material for: Implementation and effectiveness of transgender stigma reduction interventions in sub-Saharan Africa: a scoping review
Source: J Glob Health Rep. Author manuscript; Available in PMC 2024 Aug 29. (PMC11361317; doi:10.29392/001c.72080)
Supplement: Supplementary Table1 1 [file NIHMS1962309-supplement-Supplementary_Table1_1.pdf]

Supplementary Table 1: Search strategy applied to PUBMED and adapted for use in other databases

Query	Fields	Search term [MeSH]
#1	All	Transgender OR LGBTQ OR Key populations OR sexual and gender minorities OR gender identity
#2	All	Stigma OR social stigma OR discrimination
#3	All	Prevention intervention OR reduction intervention OR mitigation intervention
#4	All	sub-Saharan Africa
#5	#1AND#2 AND#3 AND#4	
